# Replicating Human Hand Synergies Onto Robotic Hands: A Review on Software and Hardware Strategies

**DOI:** 10.3389/fnbot.2018.00027

**Published:** 2018-06-07

**Authors:** Gionata Salvietti

**Affiliations:** ^1^Department of Information Engineering and Mathematics, University of Siena, Siena, Italy; ^2^Department of Advanced Robotics, Istituto Italiano di Tecnologia, Genoa, Italy

**Keywords:** human hand synergies, robotic hand control, mapping strategies, human motor control, hand

## Abstract

This review reports the principal solutions proposed in the literature to reduce the complexity of the control and of the design of robotic hands taking inspiration from the organization of the human brain. Several studies in neuroscience concerning the sensorimotor organization of the human hand proved that, despite the complexity of the hand, a few parameters can describe most of the variance in the patterns of configurations and movements. In other words, humans exploit a reduced set of parameters, known in the literature as synergies, to control their hands. In robotics, this dimensionality reduction can be achieved by coupling some of the degrees of freedom (DoFs) of the robotic hand, that results in a reduction of the needed inputs. Such coupling can be obtained at the software level, exploiting mapping algorithm to reproduce human hand organization, and at the hardware level, through either rigid or compliant physical couplings between the joints of the robotic hand. This paper reviews the main solutions proposed for both the approaches.

## 1. Introduction

In the last decade, several roboticists have tried to replicate human hand motor control to possibly simplify the design and actuation of robotic hands. The neuroscientific foundation supporting this approach is the demonstration that, despite the intricate nature of the human hand, a reduced number of variables is able to explain a large part of the variance in patterns of the human hand configurations and movements, as pioneered by Bernstein (1966) and later reported by Santello et al. (1998). These variables are usually referred to as *postural synergies* and can be interpreted as a correlation of DoFs in frequently used patterns, Santello et al. (2016). Several experimental approaches, ranging from recording of electromyography and cortical activities to the studies of finger movement kinematics, have investigated the neural control of the hand. The results confirms that the simultaneous motion of the fingers underlay to coordinated patterns that reduce the number of independent DoFs to be controlled. The idea that particular arrangements of muscular activities could compose a base set analogous to the concept of basis in the theory of vector spaces was introduced by Easton (1972). Todorov et al. (2005) proposed an optimal stochastic control based on the same geometrical system of redundancy resolution.

Santello et al. (1998) investigated the postural synergies hypothesis by recording a large data set of grasping poses from subjects that were asked to mime grasps of a set of 57 objects. A Principal Components Analysis (PCA) of this data reported that more than 80% of the variance could be accounted with the first two principal components, whereas the first three components explained up to 90% of the variance in the data. This suggests that a much lower-dimensional subspace of the hand DoFs space can efficiently characterize the recorded data. In other words, instead of controlling the single 20 DoFs of a human hand, only two or three joints coupling leading to coordinated motions of the hand could be used to achieve many of the grasps used in everyday life. These ideas can be exploited in robotics, as they introduce a novel and principled manner to simplify the design and analysis of hands different from other sometimes arbitrary and more empirical design attempts.

In this work, the main approaches used to design and control robotic hands exploiting the synergy concept are reviewed. First, several robotic hands mechanically designed to resemble human hand synergies are described in section 3. Then, in section 4, the principal mapping algorithms used to synergically control multi-DoFs hands are introduced. Finally, in section 5, a discussion on the current work and future direction is reported.

## 2. Hand synergies from neuroscience to robotics

Fully actuated robotic hands have been extensively studied and several tools for modeling and control are available in literature, as reported by Murray et al. (1994) and Prattichizzo and Trinkle (2016). However, to fully exploit the wide dexterity of multi-DoF hands with independent actuated joints it is necessary to design sophisticated control strategies that often represent the main roadblock to the plain usability and efficiency of robot hands in real-world scenarios. Among the several attempts to reduce robotic hand control parameters, the one based on synergies is attracting a critical mass of researchers. The main reason behind this diffusion resides on the neuroscientific results reporting that, between other possible choices for the basis to describe the hand configuration, most of the hand grasp posture variance is explained by the first two synergies, as reported by Santello et al. (1998).

A direct interpretation of these results would implicate that the robotic hand joint configuration vector $q\in {ℜ}^{n_{q}}$, where *n*_*q*_ is the number of joints in the hand, could be represented as a function of fewer elements, collected in a *synergy vector* $z\in {ℜ}^{n_{z}}$ with *n*_*z*_ ≤ *n*_*q*_. As formalized by Bicchi et al. (2011) and Prattichizzo et al. (2013), indicating with $\overset{\cdot }{q}$ the hand joint velocities, we can define the linear map $\overset{\cdot }{q}=S\left(z\right)ż$, where *S* is the synergy matrix and ż represents synergy velocities. Columns of the matrix of synergies $S\in {ℜ}^{n_{q}\times n_{z}}$ represent the postural synergies, also named as *eigengrasps* in the literature, e.g., by Ciocarlie and Allen (2009). In other terms, the columns represent the joint velocities that are obtained acting on each single synergy ż. This pure kinematic model fails to describe the possible grasps of an object since does not consider a possible hand adaptation to the shape of the grasped object. A possible solution is to consider the most general case of statically-indeterminate grasps (Prattichizzo and Trinkle, 2016), and thus introduce both contact and joint compliance in the analysis. Doing so, we assume that the synergistic hand displacements $\delta z\in {ℜ}^{n_{z}}$ does not directly command the joint displacements $\delta q\in {ℜ}^{n_{q}}$, but the synergistic displacements input δ*z* commands the joint reference positions *q*_*ref*_ as:

(1) $$\begin{array}{r} \delta q_{ref}=S\delta z, \end{array}$$

which are related to the actual joint displacements by the constitutive equation:

(2) $$\begin{array}{r} \delta q=\delta q_{ref}-C_{q}\delta \tau , \end{array}$$

where *C*_*q*_ models the joint compliance and δτ represents the torques at the joints, as reported by Prattichizzo et al. (2010). When no contact with the object is present, the reference and real joints positions overlap, whereas if contact forces are present, the compliance of the hand forces the real hand to diverge from the reference one. This means that the real hand configuration is synergy driven, but can modify its posture so to comply with the object shape. Gabiccini et al. (2011) defined this approach as *soft synergy* model of hands.

In the following sections, the main attempts to reproduce, either mechanically or by means of the control, the matrix *S* representing the synergistic joint coupling are reported.

## 3. Mechanical implementation of postural synergies

**Figure 1:**
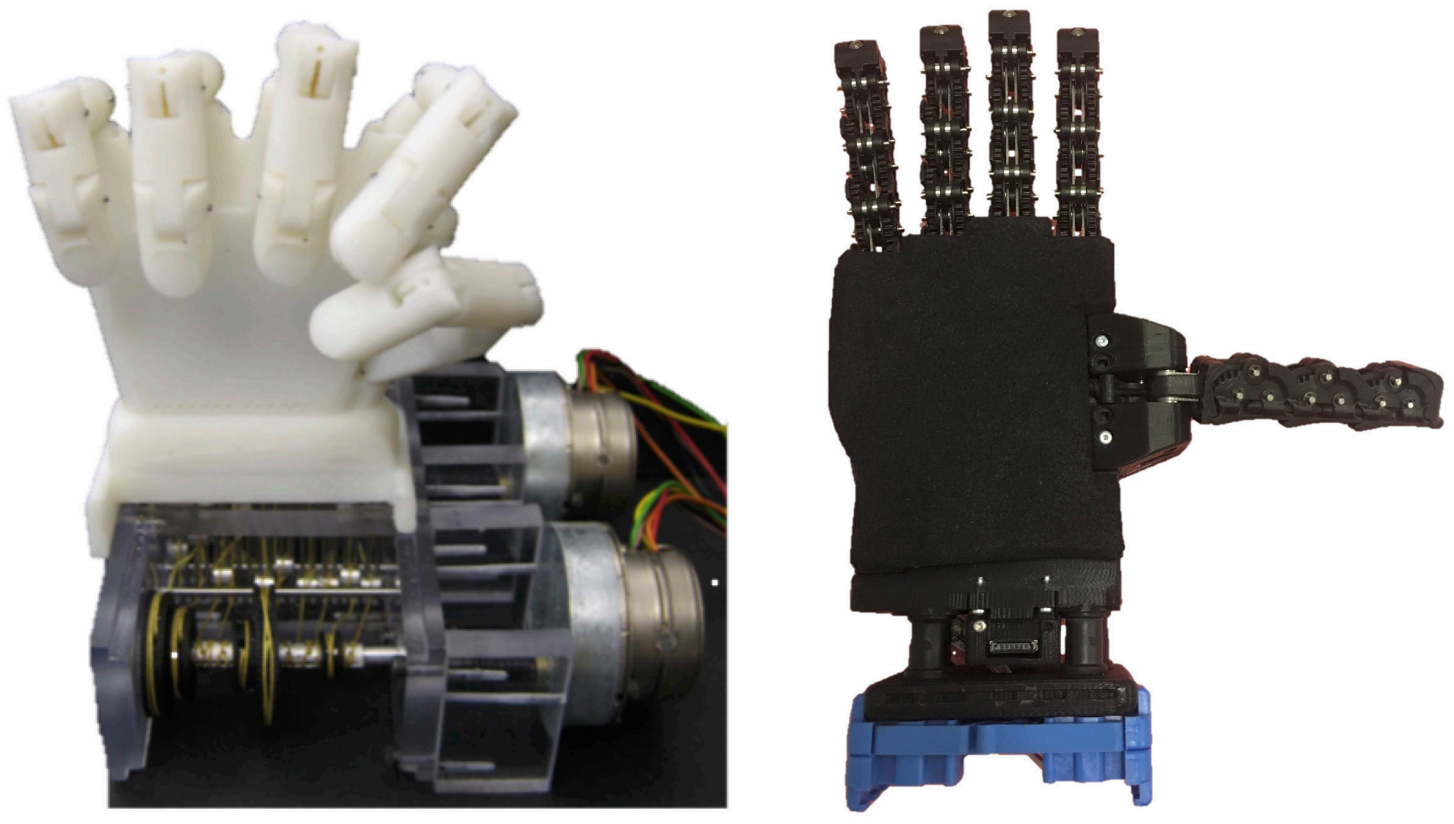
Two of prototypes of robotic hands that mechanically implement synergies. On the left, rigid synergies are obtained through a mechanism that embeds tendons and pulleys, from Brown and Asada (2007), License Number 4350150698742. On the right side, adaptive synergies are obtained designing the tendon transmission system and the joint compliance.

In section 2, two possible ways to model the hand synergies have been introduced. The distinction between “rigid” and “soft” synergies also represents the two main approaches in literature to mechanically implement the coordination of joint motions in underactuated hands. Brown and Asada (2007) pioneered the idea of using a mechanism to rigidly couple the motion of the joints according to the human synergies. A train of pulleys of different radii was used to transmit simultaneously different motions to each joint. The radii of the pulleys were set according to the scalar weight that compose the columns of synergy matrix *S*. In other words, changing the radius of the pulleys, it was possible to regulate how much a certain joint is displaced once the motor is activated. Motions corresponding to the first two synergies were superimposed via tendons and idle pulleys resulting in the prototype illustrated in the left hand side of Figure 1. A similar approach has been used by Li et al. (2014) to design a prosthetic hand where twelve DoFs are activated using only two motors. Xu et al. (2014b) proposed a prototype where the postural synergies were mechanically implemented in an underactuated anthropomorphic hand using planetary gears. Rosmarin and Asada (2008) proposed a hybrid actuation system using two DC motors and shape memory alloy (SMA) actuators. The two DC motors drove the entire robotic hand according to the direction of the two most significant synergies. The synergies were determined through the PCA analysis of a set of robotic hand postures. The higher order terms were actuated by SMA so to reduce the actuators' encumbrance.

**Figure 2:**
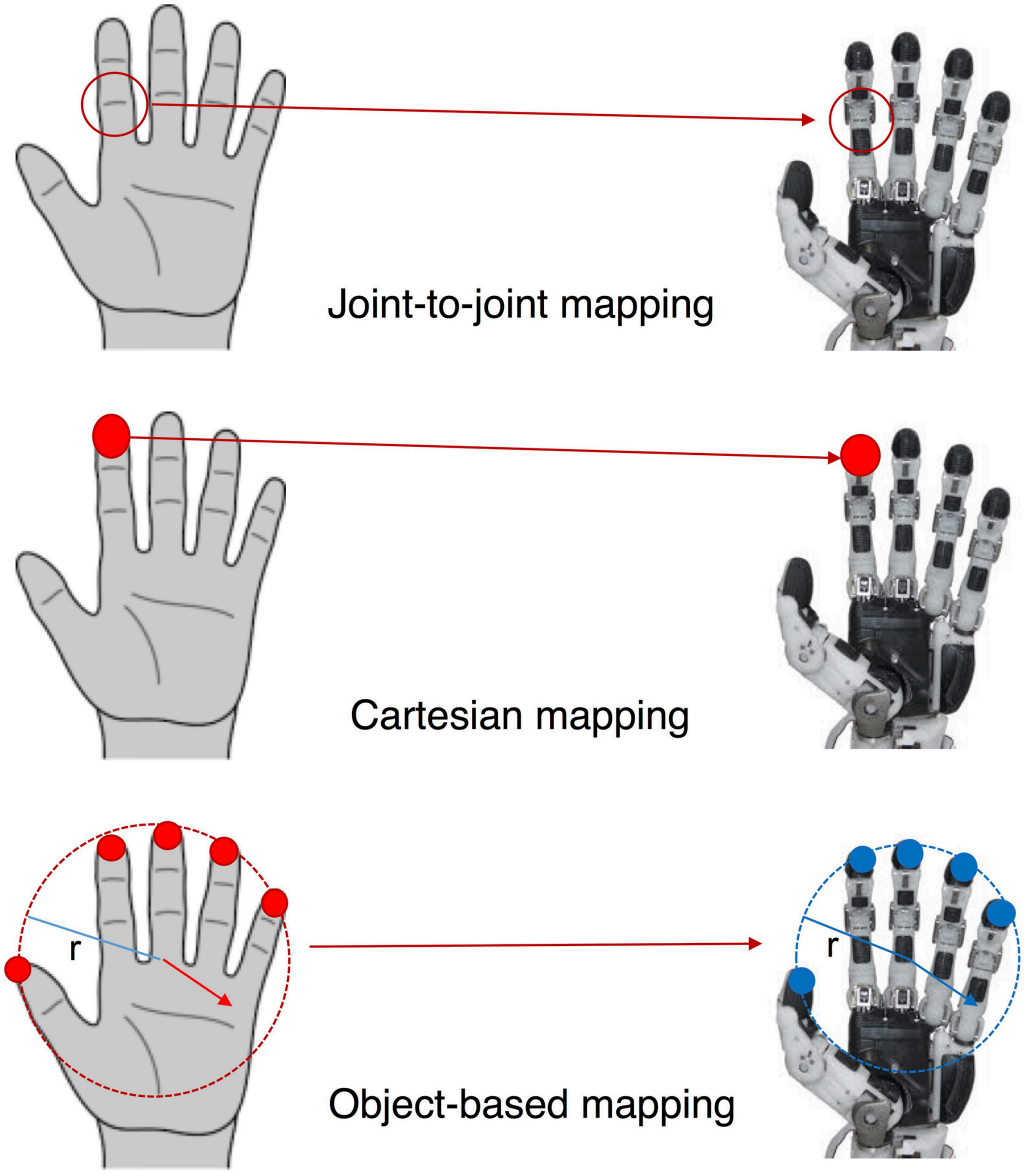
Schematic representation of the principal mapping techniques. In the top, joint-to-joint mapping. In the center, an example of Cartesian mapping where fingertips positions are mapped. In the bottom, the object-based mapping where the motions and deformations of the two virtual objects are put in correspondance.

The “soft” synergies approach described in section 2 is an efficient solution to design anthropomorphic hands with a synergistic motion. Catalano et al. (2014) have investigated how to exploit the soft synergy concept through the design of underactuated hands that have desirable adaptivity to shapes of the grasped objects. Birglen et al. (2008) have reported how underactuation can be achieved effectively with simple differential and elastic elements. Catalano et al. leveraged on this design principles to realize a soft synergy model defined by a synergy matrix *S* and a joint compliance matrix *C*_*q*_ through the definition of a proper transmission matrix and the design of the joint stiffness. The authors defined this solution as *adaptive synergies*. The resulting prototype, called the *Pisa/IIT SoftHand*, has 19 DoFs arranged in four fingers and an opposable thumb, see the right side of Figure 2. Only one actuator drives all the fingers so to resamble the first synergy defined as in Santello et al. (1998). Recently, Piazza et al. (2017) have exploited the same concept of adaptive synergies to design a prosthetic hand.

Finally, Xu et al. (2014a) proposed a continuum structure for the mechanical implementation of the postural synergies. Using a continuum mechanism, two independent translational inputs were scaled and combined to generate six translational outputs to drive a prosthetic hand prototype.

## 4. Software procedures for synergistic control of multi-dof robotic hands

Software synergies refers to all the techniques that have been proposed in literature to control a multi-DoF hand with a reduced number of parameters so to resamble the synergistic actuation of the human hand. The several approaches proposed over the last decade can be classified into two main categories: (i) mapping of synergies from humans to robots, and (ii) redefinition of synergies for robotic hands. The main idea of the former method is to define a synergy matrix computed through some statistical analysis of human poses over objects and to replicate the synergistic motion onto the kinematic of the robotic hand using a proper mapping strategy. The work of Ciocarlie and Allen (2009) is one of the first examples of this method. They used a joint-to-joint mapping to replicate the synergy subspaces obtained by Santello et al. onto four different models of hands. Joint-to-joint mapping considers a direct association between joints on the human hand and joints on the robotic hand. Other researchers have investigated this approach. Rosell and Suárez (2014) used a sensorized glove to collect data from the joints of the human hand captured while an operator was moving freely the fingers, i.e., without executing or simulating grasping or manipulation actions, and then joint-to-joint mapped the data onto the Schunk SAH hand. Kim et al. (2016) proposed an algorithm that uses a tensor composed of data relevant to different individuals and various motions in multiple dimensions to evaluate human hand synergies. The corresponding values for a robot hand were then computed assuming that the coefficients of the synergies of the human hand were identical to those of the robotic hand. It is worth noting that joint-to-joint mapping represents the simplest way to define a correspondence between the joints of human and robotic hands. This mapping results efficient when the robotic hand has an anthropomorphic structure, whereas, when non-anthropomorphic devices are considered, the joint correspondence is usually defined considering some heuristics that often reduce the reliability of the motion reproduction.

Another method to map the human hand synergies is the so called Cartesian space mapping. Cartesian mapping focuses on the relation between the workspaces of the human and robot hand and usually tries to find a correspondence between the motion of the fingertips of the two hands. Ficuciello et al. (2018) mapped human grasps onto a robotic underactuated hand using fingertips measurements, obtained through a RGBD camera sensor, and inverse kinematics. Geng et al. (2011) realized a two stage mapping. Firstly, they extracted the synergies from human grasping data and later they implemented an optimized mapping to replicate fingertip positions of the human hand to those of a robot hand. Cartesian mapping presents some advantages with respect to the joint-to-joint mapping, since it is not a necessity to relate each robotic joint motion to that of human joints. However, this method fails in replicating a correct mapping in terms of forces and movements exerted by the robotic hand on a grasped object. Gioioso et al. (2013) have presented a method for mapping synergies defined in the task space that tries to overcome the problem of dissimilar kinematics between human and robotic hand. The main idea of the approach is to define two virtual objects, one on the robotic hand and one on a model of the human hand. Each virtual object is defined considering the minimum volume sphere containing a set of reference points defined on the hand, see the bottom part of Figure 2. The human hand model can be moved according to a synergistic motion computed using the dataset of Santello et al. (1998). Such motion displaces the reference points on the hand generating a rigid body motion and a deformation of the virtual object. These transformations of the object are then imposed, possibly scaled, onto the object defined on the robotic hand. An inverse kinematic technique is used to compute robotic joint motions that comply with the virtual object motion and deformation. The authors proved that the virtual object method is more efficient in terms of force mapping and accuracy in reproduction of the directions of motion with respect to joint-to-joint and Cartesian mappings. In Gioioso et al. (2012), the method was extended considering an ellipsoid instead of a sphere as virtual object. This improvement consented to describe the virtual object deformation using three parameters, the ellipsoid semi-axis variations, instead of one, the sphere radius variation. Salvietti et al. (2014) used the average homogenous transformation of the reference points so to capture a larger set of possible motions of the virtual object. All the techniques related to the object-based mapping of the human hand synergies have been collected in a freely available Matlab toolbox, called SynGrasp (Malvezzi et al., 2015). Figure 2 shows a schematic representation of the mapping strategies.

The second main approach to define software synergies consists in collecting data from grasps obtained directly with the robotic hand and using a statistical analysis to extract the primitives for the specific hand. Ficuciello et al. (2014) computed the first two fundamental synergies for the UB Hand IV applying PCA on a set of 36 grasps of different objects, involving both precision and power grasps. Matrone et al. (2010) collected the sensory data of a prosthetic hand while performing 50 different grasps, and subsequently used a PCA based algorithmto drive the 16 DoFs of an underactuated prosthetic hand prototype, called CyberHand, with a two dimensional control input. Wimböck et al. (2011) analyzed a large grasp database collected over years of use of the DLR Hand II. Using PCA, they found that 74% of these grasps, originally defined by the twelve joint variables of the hand, could be represented by two coordinates. As a second step, a synergy impedance controller was derived and implemented to extend the work on passivity based hand control for the DLR Hand II. Later, Salvietti et al. (2013) combined the object basedmapping with the synergy impedance controller to simplify robotic hand control in the synergy subspace. Bernardino et al. (2013) teleoperated a Shadow Hand and an iCub Hand so to perform the grasp of 12 different objects and then used the collected joint data from the robotic hands to compute postural synergies using PCA. Finally, Cotugno et al. (2014) used a kinaesthetic teaching approach to collect data from the iCub Hand. The teaching was performed by a human operator guiding the fingers of the robot with the motors switched off so to perform a pick and place operation over a set of objects. Singular value decomposition was later performed on the pre-processed joint data in order to obtain the postural primitives of the hand that span the variability of the corresponding grasping demonstrations.

## 5. Discussion and perspective

In this review, the main mechanical and software solutions that explicitly exploit the concept of human hand synergies have been reported. The main reason is the direct link between neuroscientific studies and robotics. There are several other works on underactuation both from a software and a hardware point of view that have not been treated in this work. Among all, it is worth mentioning recent results on the design of underactuated soft hands which are designed to include intrinsic passive compliant elements, see e.g., the hand proposed by Deimel and Brock (2016). In this context, Salvietti et al. (2017) have proposed a procedure to compute the stiffness ratio between the passive compliant joints of a robotic hand so to resemble the trajectory for the fingertips obtained through the execution of the first synergy.

Concerning the software synergies, both the presented approaches have pros and cons. The use of data collected from the human hand allows to exploit human brain control mechanisms that resulted from thousands of years of evolution. However, the adaptation of the data to the kinematics of a robotic hand is prone to errors that may compromise the fine control of the forces exerted on a grasped object. On the contrary, synergies defined directly on the robotic hand are highly specialized for the specific hand kinematics, but may highly depend on the set of grasps decided by the operator or by the operator kineasthetic teaching. This could result in very specialized primitives that may difficultly generalize over a wider set of objects.

Although the complexity reduction brought by the synergistic organization of the hand have led to encouraging results in grasping, how to exploit high order synergies to perform more complex manipulation tasks is still an open issue. A possible tradeoff between the complexity of the control and the level of dexterity of the robotic hand will probably come from a more deep interaction between designers and controllers so to embed part of the control directly in the hand structures.

## Author contributions

GS have organized and written this mini-review. GS has also contribute to the topic of the review with publications that have been mentioned in the review.

### Conflict of interest statement

The author declares that the research was conducted in the absence of any commercial or financial relationships that could be construed as a potential conflict of interest.
